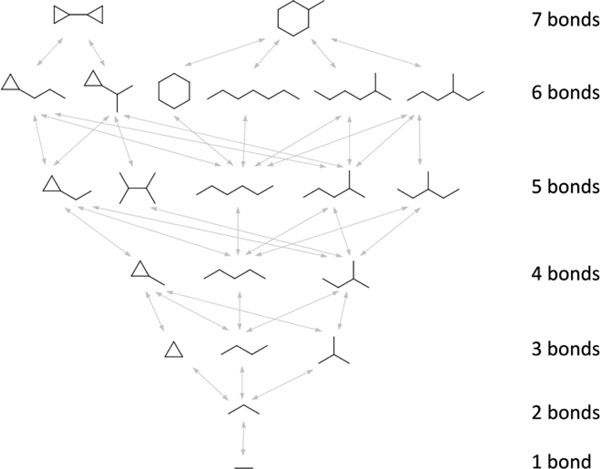# Efficient maximum common subgraph (MCS) searching of large chemical databases

**DOI:** 10.1186/1758-2946-5-S1-O15

**Published:** 2013-03-22

**Authors:** Roger A Sayle, Jose Batista, J Andrew Grant

**Affiliations:** 1 NextMove Software Limited, Cambridge, Cambridgeshire, CB4 0EY, UK; 2 Discovery Sciences, AstraZeneca R&D, Alderley Park, Cheshire, SK10, UK

## 

Despite dramatic improvements in the hardware resources and computational power available to pharmaceutical researchers over the past few decades, the methods used for assessing the 2D chemical similarity between two molecules hasn't changed much since the 1960s. Here we report a novel chemical database search method that allows the exact size of the maximum common edge subgraph (MCES) between a query molecule and molecules in a database to be calculated rapidly. Using a pre-computed index, the 50 nearest neighbors of a query can be determined in a few seconds, even for databases containing millions of compounds. This work builds upon the previous efforts of Wipke and Rogers in the 1980s [1] and of Messmer and Bunke in the 1990s [2], harnessing the advances in high-performance computing and storage technology now available. A graphical depiction of such a "SmallWorld" index is shown below.

**Figure 1:**